# Exploring Erenumab’s Efficacy and Safety for Migraine Prevention in Real-World Settings: A Systematic Review

**DOI:** 10.7759/cureus.65571

**Published:** 2024-07-28

**Authors:** Mah Rukh Nisar, Rudrani Kotha, Sabaa I Saad-Omer, Shivani Singh, Oluwatoba T Olayinka, Jaslin Orelus, Ann Kashmer Yu

**Affiliations:** 1 Neurology, California Institute of Behavioral Neurosciences and Psychology, Fairfield, USA; 2 Internal Medicine, California Institute of Behavioral Neurosciences and Psychology, Fairfield, USA; 3 Clinical Sciences, California Institute of Behavioral Neurosciences and Psychology, Fairfield, USA; 4 Emergency Medicine, California Institute of Behavioral Neurosciences and Psychology, Fairfield, USA

**Keywords:** anti-cgrp mab, treatment resistant migraine, episodic migraine, chronic migraine, migraine, erenumab

## Abstract

Migraine causes debilitating headaches and significantly impacts quality of life. Effective migraine-specific treatments have been lacking until the advent of monoclonal antibodies (mAbs) targeting calcitonin gene-related peptide (CGRP) receptors, which have expanded therapy options for migraine treatment. This study explores the short- and long-term efficacy and safety of erenumab in migraine treatment. The Preferred Reporting Items for Systematic Reviews and Meta-Analyses (PRISMA) 2020 criteria guided this systematic review. Five databases - PubMed, PubMed Central, Google Scholar, ScienceDirect, and Sage Journal - were searched for published, freely accessible, full-text articles in English from the past five years. Eligible patients included those with episodic or chronic migraines who received erenumab intervention. From an initial search yielding 680 relevant studies, 12 prospective observational cohort studies were selected after assessing the risk of bias through the Quality Assessment Tool for Observational Cohort and Cross-Sectional Studies. All included studies demonstrated a significant reduction in monthly migraine days (MMDs) by the end of the treatment period, with mild adverse effects observed. No significant short-term or long-term safety concerns were identified.

## Introduction and background

Migraine, a debilitating neurological condition, causes frequent episodes of headache that are accompanied by elevated sensitivity and signs of parasympathetic dysfunction [1]. One of the aspects that defines an episodic migraine (EM) is the pain phase; apart from that, the prodromal, aura, and postdromal stages are frequently present throughout the migraine cycle [2]. At least 15 headache days per month, eight of those days being migraine days, is considered chronic migraine (CM) [3]. Considering the frequency of migraines, all CM patients are eligible for preventive treatment [3].

Pharmaceutical medications like topiramate, valproate, β-blockers, and amitriptyline, which were not specifically designed for migraine therapy, are commonly used in the preventive treatment of migraine [4]. Due to ineffectiveness or poor tolerability, these non-specific treatments have a high dropout rate [4]. Since calcitonin gene-related peptide (CGRP) is important to the pathogenesis of migraines, targeting the CGRP receptor is pertinent for migraine prevention [5]. The human body contains the neuropeptide CGRP, which is mostly concentrated in the trigeminovascular system; blood, tears, saliva, and cerebrospinal fluid all have higher CGRP levels during an EM, and these levels return to normal following the attack [6]. Additionally, CGRP levels are permanently raised during CM [6].

With the advent of monoclonal antibodies (mAbs) that target CGRP or its receptor, the therapy options for migraine prevention have increased, offering those with "refractory" migraines new hope [7]. In clinical trials, these therapies have outperformed placebo in terms of lowering mean monthly migraine days (MMDs) and enhancing migraine-related quality of life measures for all migraine subtypes [7]. A fully human mAb that targets the CGRP receptor is erenumab, the first of its kind [8]. Three to six randomized controlled trials (RCTs) showed that erenumab-treated migraine patients experienced a statistically significant decrease in MMDs, monthly headache days (MHDs), and monthly migraine-specific medication days (MSMDs) [8].

In both EM and CM, the effectiveness, acceptability, and safety of erenumab 70 mg and 140 mg given once a month by subcutaneous injection have been proven [9]. As a result, these are the two commercially available dosages for migraine prophylaxis [10]. Cardiovascular safety is the primary issue when employing mAbs that target the CGRP pathway, as these antibodies express the CGRP receptor on smooth muscle cells in arteries and impede natural vasodilation through the CGRP-CGRP receptor connection [10]. Numerous preclinical investigations demonstrated that the therapy mostly affects the distal coronary arteries, meaning that people without coronary artery disease are not at risk [10]. This systematic review has been conducted to investigate the short- and long-term efficacy and safety of erenumab in migraine patients in prospective real-world studies.

## Review

Methods

The Preferred Reporting Items for Systematic Reviews and Meta-Analyses (PRISMA) 2020 criteria served as the basis for this systematic review [11].

Eligibility Criteria

The eligibility criteria for inclusion in the analysis required that studies involve patients diagnosed with CM or EM according to the International Classification of Headache Disorders, 3rd edition (ICHD-3) criteria. The studies needed to focus on the use of erenumab as the intervention. Additionally, only published, freely accessible, full-text articles in English from the last five years that were prospective observational studies were considered.

Literature Search

Five databases, including PubMed, PubMed Central, Google Scholar, ScienceDirect, and Sage Journals, as well as grey literature, were searched. The last date of search for all databases was March 28. Table 1 illustrates how the process's field search was chosen using Medical Subject Headings (MeSH) and keywords from earlier research, depending on the database. 

**Table 1:** Literature search strategy CGRP: Calcitonin gene-related peptide

Databases	Keywords	Search strategy	Filters	Search results
PubMed	Erenumab OR anti-CGRP monoclonal antibody OR Chronic Migraine OR Migraine	CONCEPT 1: ERENUMAB OR anti-CGRP monoclonal antibody OR ("Receptors, Calcitonin Gene-Related Peptide/drug effects"[Majr] OR "Receptors, Calcitonin Gene-Related Peptide/therapeutic use"[Majr]) CONCEPT 2: CHRONIC MIGRAINE OR MIGRAINE PREVENTION OR ("Migraine Disorders/drug therapy"[Majr] OR "Migraine Disorders/prevention and control"[Majr] OR "Migraine Disorders/therapy"[Majr]) CONCEPT 1 AND 2: ERENUMAB OR anti-CGRP monoclonal antibody OR ("Receptors, Calcitonin Gene-Related Peptide/drug effects"[Majr] OR "Receptors, Calcitonin Gene-Related Peptide/therapeutic use"[Majr]) AND CHRONIC MIGRAINE OR MIGRAINE PREVENTION OR ("Migraine Disorders/drug therapy"[Majr] OR "Migraine Disorders/prevention and control"[Majr] OR "Migraine Disorders/therapy"[Majr])	Free full text In the last five years, English	317
PubMed Central	Erenumab, migraine	Erenumab AND Migraine	2020 to 2024, Research articles	100
Google Scholar	Erenumab, migraine	Erenumab AND Migraine (all in title)	2020 T0 2024, All articles, English	160
Science Direct	Erenumab, migraine	Erenumab AND Migraine	2020 2024, research articles, English; Open access and open archive	23
Sage Journals	Erenumab, migraine	Erenumab AND Migraine	2020 to 2024, research articles	80

Study Selection

After the initial search of databases, duplicates were removed. Initial screening was based on titles and abstracts; all irrelevant studies were removed. Based on the inclusion criteria, only 15 studies were selected, and three were removed after quality assessment. Thus, 12 prospective observational cohort studies were selected for the final review. Two authors screened the articles independently.

Risk of Bias in Individual Studies

The Quality Assessment Tool for Observational Cohort and Cross-Sectional Studies Risk Bias Tool was used for risk of bias assessment [12]. Twelve good-quality studies, with greater than 70% quality assessment percentage, were selected, as shown in Table 2. 

**Table 2:** Quality assessment of individual studies Y: Yes; N: No; NA: Not applicable

Assessment criteria	Lambru et al. (2020) [13]	Ornello et al. (2020) [14]	Ornello et al. (2020) [15]	Russo et al. (2020) [16]	Tziakouri et al. (2021) [17]	de Vries Lentsch et al. (2021) [18]	Ornello et al. (2022) [19]	Andreou et al. (2022) [7]	Bacas et al. (2022) [20]	Becker et al. (2022) [8]	Cullum et al. (2022) [21]	Lanteri-Minet et al. (2023) [22]
Did the paper clearly articulate its research question or objective?	Y	Y	Y	Y	Y	Y	Y	Y	Y	Y	Y	Y
Was the study population clearly identified and described?	Y	Y	Y	Y	Y	Y	Y	Y	Y	Y	Y	Y
Did at least 50% of eligible participants take part in the study?	Y	Y	Y	Y	Y	Y	Y	Y	Y	Y	Y	Y
Were subjects consistently selected from similar populations and time periods? Were inclusion and exclusion criteria uniformly applied to all participants?	Y	Y	Y	Y	Y	Y	Y	Y	Y	Y	Y	Y
Was a justification for the sample size, including power analysis or estimates of variance and effect, provided?	Y	Y	Y	Y	Y	Y	Y	Y	N	N	N	Y
Were the exposures measured before the outcomes in this study?	Y	Y	Y	Y	Y	Y	Y	Y	Y	Y	Y	Y
Was the timeframe adequate to reasonably detect an association between exposure and outcome?	Y	Y	Y	Y	Y	Y	Y	Y	Y	Y	Y	Y
Did the study analyze different levels of exposure in relation to the outcome?	Y	Y	Y	Y	Y	Y	N	Y	Y	Y	Y	Y
Were the exposure metrics well specified, accurate, reliable, and used uniformly across all subjects?	Y	Y	Y	Y	Y	Y	Y	Y	Y	Y	Y	Y
Was the exposure evaluated multiple times throughout the study duration?	Y	Y	Y	Y	Y	Y	Y	Y	Y	Y	Y	Y
Were the result metrics properly stated, reliable, valid, and used uniformly across all subjects?	Y	Y	Y	Y	Y	Y	Y	Y	Y	Y	Y	Y
Were those assessing the outcomes unaware of the participants' exposure status?	NA	NA	NA	NA	NA	NA	NA	NA	NA	NA	NA	NA
Was the loss to follow-up after baseline 20% or lower?	N	Y	N	Y	Y	Y	Y	N	N	N	N	N
Were significant potential confounding factors measured and statistically adjusted for their impact on the association between exposure and outcome?	NA	NA	NA	NA	NA	NA	NA	NA	NA	NA	NA	NA
Accepted score ≥70%	11/14 (78%)	12/14 (85.7%)	11/14 (78%)	12/14 (85.7%)	12/14 (85.7%)	12/14 (85.7%)	11/14 (78%)	11/14 (78%)	10/14 (71%)	10/14 (71%)	10/14 (71%)	11/14 (78%)

Results

**Figure 1:**
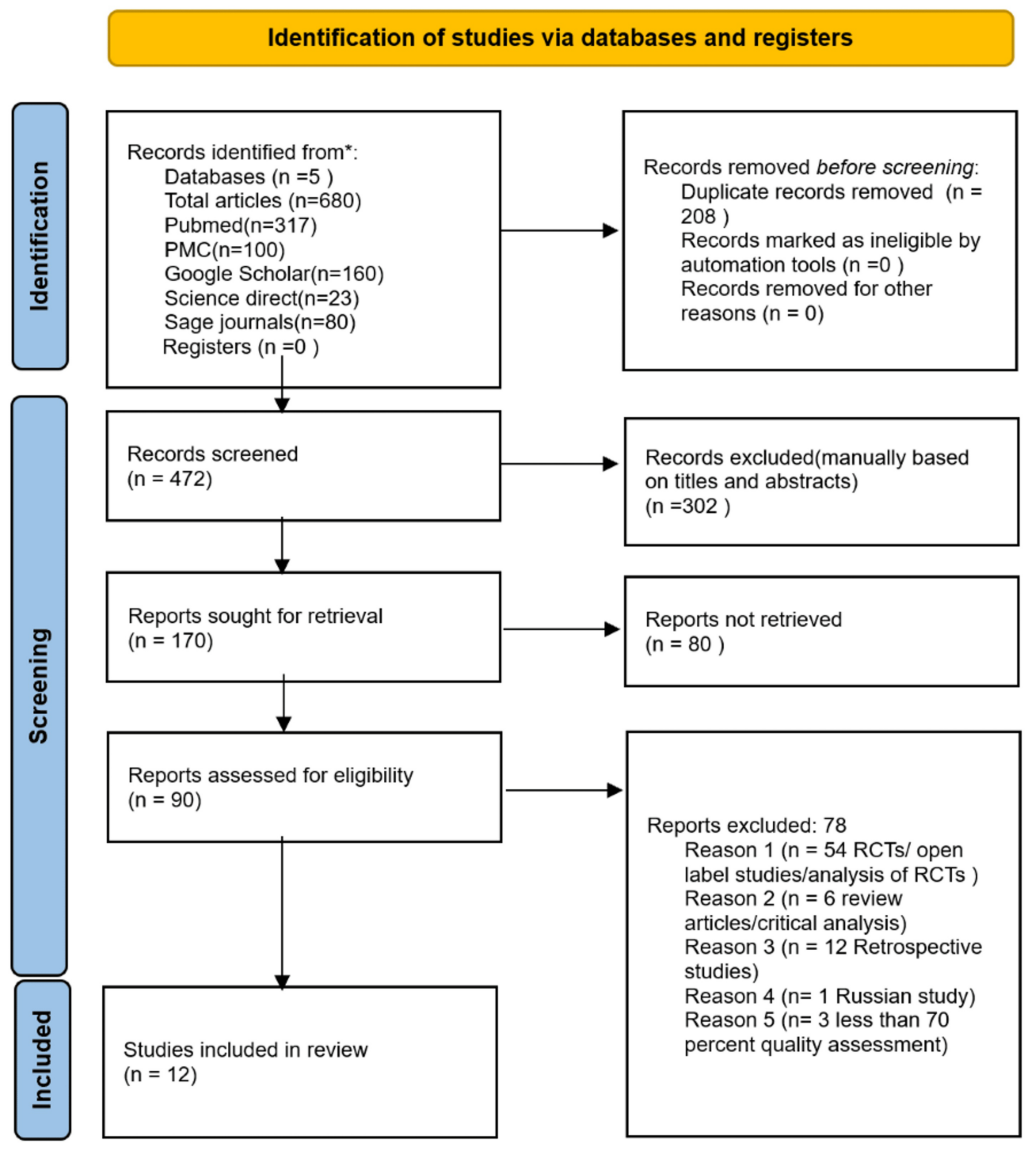
Flow diagram for screening and selection process RCTs: Randomized controlled trials

After the initial search of databases, there were a total of 680 relevant studies, with 472 remaining after duplicate removal. Initial screening was based on titles and abstracts, and all irrelevant studies were removed. Based on the inclusion criteria, only 15 studies were selected, two of which were removed after quality assessment, resulting in 12 prospective observational cohort studies being selected for final review, with a score of greater than 70%. The flow diagram in Figure 1 demonstrates the screening and selection process. 

Study Characteristics

A total of 12 prospective cohort studies included 2,427 patients given at least one erenumab injection. Among these, 2,094 were females and 503 were males. Patients received erenumab at either 70 mg or 140 mg, with some starting at 70 mg and increasing to 140 mg if they did not respond initially. The studies evaluated outcomes such as MMDs, MHDs, headache impact test (HIT) scores, and migraine disability assessment (MIDAS) scores, as well as the safety of erenumab. Table 3 provides the characteristics of these studies.

**Table 3:** Characteristics of individual studies MMDs: Monthly migraine days; MHDs: Monthly headache days; HIT-6: 6-item headache impact test, HR QOL: Health-related quality of life; EM: Episodic migraine; CM: Chronic migraine; CONV: Conversion; F: Female; M: Male

Study	Number of patients	Year of study	Previous treatment failures	Gender	Total follow-up period	Outcomes studied	Treatment discontinuation
Lambru et al. (2020) [13]	162	2020	≥3	F (135)/M (27)	6 months	MMD, MHD, HIT-6	40%
Ornello et al. (2020) [14]	91	2020	≥2	F (80)/M (49)	6 months	MHD, CM CONV TO EM	12.1%
Ornello et al. (2020) [15]	89	2020	≥2	F (78)/M (11)	6 months	MMD, MIDAS, HIT-6	32.6%
Russo et al. 2020 [16]	70	2022	≥4	F (55)/M (15)	6 months	MHD, CM CONV TO EM, MIDAS, HIT6	0%
Tziakouri et al. 2021 [17]	16	2021	≥3	F (14)/M (2)	6 months	MMD, HR QOL	13%
de Vries Lentsch et al. (2021) [18]	100	2021	≥4	F (85)/M (15)	6 months	MMD	5%
Ornello et al. (2022) [19]	1175	2022	None	F (985)/M (230)	3 months	MMD, HIT6	2.8%
Andreou et al. (2022) [7]	160	2022	≥3	F (132)/M (28)	24 months	MMD, HIT6	54%
Bacas et al. (2022) [20]	31	2022	Not given	F (22)/M (9)	12 months	MMD, MHD	74%
Becker et al. (2022) [8]	95	2022	≥2	F (76)/M (19)	6 months	MMD, MHD	24%
Cullum et al. (2022) [21]	300	2022	Not given	F (257)/M (43)	12 months	MMD, MHD	60%
Lanteri-Minet et al. (2023) [22]	140	2023	Not given	F (115)/M (25)	12 months	MMD, MHD, HIT-6	28%

Study Outcomes

The primary and secondary study outcomes, including MMDs, MHDs, HIT-6 scores, and MIDAS scores, are provided in Table 4.

**Table 4:** Study outcomes MMDs: Monthly migraine days; MHDs: Monthly headache days; MIDAS: Migraine disability assessment; HIT-6: 6-item headache impact test

Author and year	Outcomes			
	MMD	MHD	MIDAS	HIT-6
Lambru et al. (2020) [13]	Mean reduction in MMD at month 3 was 6.0 days and at month 6 was 7.5 days	Mean reduction in MHD was 6.3 days at month 3 and 6.8 days at month 6	-	Mean reduction in HIT-6 score was 7.7 points at month 3 and 7.5 points at month 6
Ornello et al. (2020) [14]	-	At months 4-6, median MHD decreased from 26.5 to 7.5 compared with the baseline in the overall group	-	-
Ornello et al. (2020) [15]	Decrease in the median number of MMDs was from 19 to 4	-	MIDAS score baseline decreased from 46 to 38 at month 6	HIT-6 score of 66 from baseline changed to 55.5 at month 6
Russo et al. (2020) [16]	-	Mean MHD at baseline 21.1 changed to 11.4 and 8.9 at months 3 and 6, respectively	Mean MIDAS score at baseline 108.1 changed to 54.5 and 51 at months 3 and 6, respectively	HIT-6 score at baseline 65.9 changed to 60.7 and 59.5 at months 3 and 6, respectively
Tziakouri et al. (2021) [17]	Baseline MMD 22 changed to 13 after erenumab treatment	-	-	-
de Vries Lentsch et al. (2021) [18]	Baseline mean MMD 14 changed to 10.2 and 9.2 at months 3 and 6, respectively	-	-	-
Ornello et al. (2022) [19]	Median MMDs decreased from 14 to 7 in the overall group	-	-	Median HIT-6 score decreased from 67 to 60 in the overall group
Becker et al. (2022) [8]	A reduction of 4.9 MMDs at week 12 and 5.7 MMDs at week 24 from baseline 15.7 MMDs	A mean MHD reduction of 4.9 and 6.1 at weeks 12 and 24, respectively	-	-
Bacas et al. (2022) [20]	Baseline MMD 13.2 changed to 8.1 at month 3 and 6.4 at month 6	Baseline MHD 18.5 changed to 13.8 at month 3 and 11.3 at month 6	Mean MIDAS score at baseline 110.1 changed to 44.6, 34.3, 21, and 21.8 at months 3, 6, 9, and 12, respectively on the MIDAS scale	HIT-6 score at baseline 67.7 changed to 59.3, 57, 53.5, and 58.8 at months 3, 6, 9, and 12, respectively
Cullum et al. (2022) [21]	Change in mean MMDs from baseline to weeks 41-52 was -9.5 days on 140-mg erenumab and -9.3 days on 70-mg erenumab	Change in mean MHDs from baseline to weeks 41-52 was -11.8 days on 140-mg erenumab and -11.8 days on 70-mg erenumab	-	-
Andreou et al. (2022) [7]	Mean reduction in MMD at month 3 was 6.0 days. At month 6, the mean reduction in MMDs for all patients was 7.5 days	-	-	Compared to the baseline, the mean reduction of HIT-6 score was 7.7 points at month 3
Lanteri-Minet et al. (2023) [22]	MMDs at baseline 19.6 changed to 11.5, 10.0, 9.2, and 9.0 at months 3, 6, 9, and 12, respectively	MHDs at baseline 22.5 changed to 14.5, 13.4, 12.2, and 11.9 months 3, 6, 9, and 12, respectively	-	HIT-6 score at baseline 68.0 changed to 59, 57, 56.5, and 56.5 at months 3, 6, 9, and 12, respectively

Discussion

This section explores the efficacy and safety of erenumab in 2,427 patients across 12 prospective cohort studies. Our analysis focuses on several key metrics: the reduction in MMDs, MHDs, HIT-6 scores, MIDAS scores, and overall health-related quality of life. The studies encompass EM and CM patients and follow their progress over varying durations: three months, six months, 12 months (in three studies), and two years (in one study). Notably, a significant proportion of the participants are women, reflecting the well-documented demographic prevalence of migraine among women. Across all studies, a consistent dosage of 70 mg of erenumab is administered, with adjustments made to 140 mg for non-responders [13]. This standardized approach ensures uniformity in treatment protocol and facilitates the comparison of outcomes across the studies. An overarching finding across all studies is at least a 30% reduction in MMDs in treatment responders. This outcome underscores the efficacy of erenumab in managing migraine symptoms over the short and long term. Furthermore, the observation of decreased migraine days aligns with previous research indicating the effectiveness of CGRP receptor antagonists in migraine management, to which erenumab belongs.

Efficacy of Erenumab

In most of the studies, following the beginning of therapy, MMD and MHD considerably decreased compared to baseline, as did the total number of days required for abortive therapy intake [13]. During the follow-up period, a significant number of patients with medication overuse discontinued the overused medication [15]. Furthermore, the HIT-6 score, which is a tool used to measure the impact of headaches on a person's daily life, decreased dramatically throughout the monitoring period in the Lambru et al. study [13]. The findings from the real-world Canadian MAGIC study also indicate that erenumab treatment was safe and achieved a ≥50% reduction in MMDs in 33.7% of participants with CM and EM who had previously experienced repeated ineffective prophylactic migraine therapies [8]. In patients with a ≥75% response rate, the residual migraine burden was minimal, with all individuals experiencing fewer than eight residual MMDs, and 74.2% having only zero to three residual MMDs [19]. In another study, among the 49 patients deemed "responders" after the third monthly subcutaneous administration of 70 mg erenumab, 92% maintained their response after the sixth administration [18]. Additionally, 57% and 62% of patients transitioned from medication overuse to non-medication overuse after the third and sixth erenumab administrations, respectively [16]. A secondary analysis of 54 patients who previously did not respond to onabotulinumtoxinA revealed that 56% of patients (30 patients) experienced a ≥30% reduction in MHDs following the sixth administration of erenumab [16]. There was no statistically significant difference observed when comparing responder rates at months 6, 9, and 12 with those at month 3 [22].

A two-year study discusses the long-term efficacy of erenumab, where, at the 12-month mark, 38% of patients sustained a minimum 30% reduction in MMDs, 26% achieved a minimum 50% reduction, and 13% experienced at least a 75% reduction in MMDs [7]. At the 24-month assessment, 23% sustained a minimum 30% reduction in MMDs, while 16% reported a minimum 50% reduction, and 8% maintained at least a 75% reduction in their MMDs [7]. In another study, out of the patients who demonstrated a ≥50% response to at least one of the initial three doses, 67.9% sustained this response through all subsequent doses [15]. In an open-label randomized trial, the long-term efficacy of erenumab was maintained for up to a year, without any safety concerns [23].

Conversion From CM to EM

In an Italian study, between months 4 and 6, 68.1% of patients transitioned from CM to EM [14]. The number of monthly converters rose from 48.4% at month 1 to 71.4% at month 5 [14]. During months 4 to 6, 16.5% of patients reached the status of low-frequency EM, 28.6% attained medium-frequency EM, and 23.1% reached high-frequency EM status [14]. In another study, 27% of refractory CM patients experienced a shift from a CM pattern to an episodic pattern following a single administration of erenumab 70 mg [13].

Safety and Tolerability

The studies reveal a concerning trend of treatment discontinuation, primarily attributed to ineffectiveness or adverse effects [13,14]. The most common mild adverse effects were constipation, fatigue, nausea, flu-like symptoms, and injection site reactions [16-18,21]. The most common adverse event that led to treatment discontinuation was constipation [15,22]. Other causes that led to treatment discontinuation were severe headaches after erenumab injections, severe flu-like symptoms, severe mood deterioration, and new-onset hypertension [13]. One patient had an allergic reaction/whole-body itchiness that was resolved after treatment was discontinued [3,15]. In some studies, erenumab demonstrated good tolerability as a treatment, with no patients discontinuing treatment due to adverse effects [6,17]. In certain studies, some patients became pregnant after receiving a few doses of the treatment, leading to the discontinuation of their treatment; however, these pregnancies proceeded without any complications [7,13,22]. In a five-year open-label extension of an RCT, the exposure-adjusted patient incidence rates of serious adverse events (SAEs) were not higher compared to the placebo or erenumab rates from the double-blind treatment phase (DBTP) pooled analysis [24]. Generally, SAEs were isolated occurrences, without a distinct treatment-related pattern [24]. In another study, no SAEs were noted in the erenumab-treated group throughout DBTP [25]. There were no fatalities documented during the study period [23]. Additionally, no clinically noteworthy alterations in laboratory parameters, or vital signs were detected throughout the open-label treatment phase (OLTP) [23].

Limitations

The eligibility criteria for this analysis, which included only published, freely accessible, full-text articles in English from the last five years, focusing on the use of erenumab in patients diagnosed with CM or EM according to the ICHD-3 criteria, introduce several limitations. These criteria result in publication bias, as studies with positive findings are more likely to be published and freely accessible, while significant studies in subscription-based journals may be excluded. Language bias arises from the exclusion of non-English research, potentially overlooking key findings from non-English-speaking countries. Temporal bias restricts the analysis of recent studies, excluding valuable older research. The focus on prospective observational studies excludes other informative designs, such as RCTs, and meta-analyses. Moreover, with only one study spanning two years, there is a need for more long-term studies to assess erenumab's long-term efficacy and safety.

## Conclusions

Our study explored the efficacy and safety of erenumab, through real-world observational studies, assessing both short-term and long-term outcomes. All reviewed studies consistently report a significant reduction in MMDs at the end of the treatment period. The findings demonstrate that erenumab significantly reduces MMDs, effectively manages migraine symptoms, and maintains a favorable safety profile. Additionally, there is notable evidence of conversion from CM to EM, as well as a decrease in the use of abortive migraine medications. Notably, patients who became pregnant during or after treatment experienced no adverse fetal or maternal pregnancy outcomes. The side effects of erenumab are generally mild, with very few adverse effects reported. Overall, erenumab emerges as a highly effective treatment option for patients with CM and treatment-resistant migraine, offering substantial benefits in terms of reduced migraine frequency and improved quality of life. Future research should continue to build on these findings to optimize its use, and further understand its long-term benefits and potential risks.

This study is crucial as it evaluates the real-world effectiveness and safety of erenumab, providing valuable insights for clinicians treating patients with treatment-resistant migraines. By analyzing both short-term and long-term outcomes, it fills a significant gap in understanding how erenumab performs outside controlled clinical trials. This research informs clinical decisions and enhances our knowledge of migraine management strategies, benefiting patient care, and guiding future research endeavors.
